# Downregulation of Soluble Guanylate Cyclase and Protein Kinase G With Upregulated ROCK2 in the Pulmonary Artery Leads to Thromboxane A2 Sensitization in Monocrotaline-Induced Pulmonary Hypertensive Rats

**DOI:** 10.3389/fphys.2021.624967

**Published:** 2021-02-03

**Authors:** Suhan Cho, Hyun Namgoong, Hae Jin Kim, Rany Vorn, Hae Young Yoo, Sung Joon Kim

**Affiliations:** ^1^Department of Physiology, College of Medicine, Seoul National University, Seoul, South Korea; ^2^Ischemic/Hypoxic Disease Institute, College of Medicine, Seoul National University, Seoul, South Korea; ^3^Department of Nursing, Chung-Ang University, Seoul, South Korea

**Keywords:** pulmonary hypertension, pulmonary artery smooth muscle, guanylate cyclase, protein kinase G, thromboxane A2 (TXA_2_), monocrotaline

## Abstract

Thromboxane A2 (TXA_2_) promotes various physiological responses including pulmonary artery (PA) contraction, and pathophysiological implications have been suggested in cardiovascular diseases including pulmonary hypertension. Here, we investigated the role of TXA_2_ receptor (TP)-mediated signaling in the pathophysiology of pulmonary arterial hypertension (PAH). The sensitivity of PA to the contractile agonist could be set by relaxing signals such as the nitric oxide (NO), soluble guanylate cyclase (sGC), and cGMP-dependent kinase (PKG) pathways. Changes in the TP agonist (U46619)-induced PA contraction and its modulation by NO/cGMP signaling were analyzed in a monocrotaline-induced PAH rat model (PAH-MCT). In the myograph study, PA from PAH-MCT showed higher responsiveness to U46619, that is decreased EC_50_. Immunoblot analysis revealed a lower expression of eNOS, sGC, and PKG, while there was a higher expression of RhoA-dependent kinase 2 (ROCK2) in the PA from PAH-MCT than in the control. In PAH-MCT, the higher sensitivity to U46619 was reversed by 8-Br-cGMP, a membrane-permeable cGMP analog, but not by the NO donor, sodium nitroprusside (SNP 30 μM). In contrast, in the control PA, inhibition of sGC by its inhibitor (1H− [1,2,4] oxadiazolo [4,3−a] quinoxalin-1-one (ODQ), 10 μM) lowered the threshold of U46619-induced contraction. In the presence of ODQ, SNP treatment had no effect whereas the addition of 8-Br-cGMP lowered the sensitivity to U46619. The inhibition of ROCK by Y-27632 attenuated the sensitivity to U46619 in both control and PAH-MCT. The study suggests that the attenuation of NO/cGMP signaling and the upregulation of ROCK2 increase the sensitivity to TXA_2_ in the PAH animal, which might have pathophysiological implications in patients with PAH.

## Introduction

Thromboxane A2 (TXA_2_), a metabolic product of arachidonic acid, is involved in various physiological activities such as platelet aggregation, airway narrowing, and contraction of various types of arteries, including the pulmonary artery (PA). TXA_2_ is synthesized by platelets and parenchymal cells of the intestine, kidney, and lung. In the pulmonary circulation system, TXA_2_ appears to act as a physiological modulator of blood flow distribution. For instance, pretreatment with TXA_2_ greatly facilitates hypoxic pulmonary vasoconstriction *in vitro* (Park et al., 2012; Yoo et al., 2012) and *in vivo* (Kylhammar and Rådegran, 2012).

Due to the short half-life (<30 s) of TXA_2_, a stable synthetic analog U46619 is widely used to investigate its physiological and pathophysiological roles (Hamberg et al., 1975; Nakahata, 2008). TXA_2_ and U46619 act through the thromboxane-prostanoid (TP) receptor which is a G-protein coupled receptor. The plasma TXA_2_ levels correlated with the prevalence of cardiovascular diseases, including hypertension (Francois et al., 2004), atherosclerosis (Mehta et al., 1988), ischemic heart disease (Serneri et al., 1981), and stroke (Stier et al., 1988). In addition, increased TXA_2_ activity was observed in pulmonary hypertension (Zamora et al., 1993).

Against the contractile signals in vascular smooth muscle cells (VSMCs) via phospholipase C (PLC)-coupled G-protein coupled receptors (GPCR) and voltage-operated L-type Ca^2+^ channels (VOCC_L_), the tone of arteries is regulated by endothelium-derived relaxing factors (EDRFs) and endothelium-derived hyperpolarizing (EDH) mechanisms. Among EDRFs, the release of NO potently regulates vascular tone through the soluble guanylate cyclase (sGC)/cGMP/protein kinase G (PKG) pathway in VSMCs (Buvinic and Huidobro-Toro, 2001). The production of NO via an endothelial type of NO synthase (eNOS) occurs primarily through an increase in cytosolic Ca^2+^ concentration ([Ca^2+^]_c_) in the endothelium and Akt-dependent phosphorylation of eNOS (Zhao et al., 2015).

The stimulation of the TP receptor in vascular smooth muscle increases [Ca^2+^]_c_ via both stored Ca^2+^ release via PLC/IP_3_ signaling and VOCC_L_-dependent Ca^2+^ influx mechanisms including the activation of non-selective cation channels (Dorn and Becker, 1993; Nakahata, 2008; Yoo et al., 2012). In addition to the direct contractile signals to VSMCs, the stimulation of TP receptors in endothelial cells inhibited K^+^ channels (e.g., SK_Ca_) through an unknown mechanism, and thus reduced the EDH signals (Ellinsworth et al., 2014). Gα_12/13_-dependent RhoA-mediated activation of kinase (ROCK) is activated by TP receptor-mediated signals that produce vascular contraction (Kozasa et al., 1998). The activation of ROCK increases the Ca^2+^-sensitivity of smooth muscle via inhibition of myosin light chain phosphatase (MLCP) (Butler et al., 2013). The U46619-induced arterial contraction was significantly attenuated by Y-27632, an inhibitor of ROCK (Fu et al., 1998). In addition to contractile signaling, our recent study suggested that TP receptor-mediated stimulation of eNOS in the PA smooth muscle layer, counterbalances the potent vasoconstrictive effect of TXA_2_ via the NO/cGMP-dependent signaling pathway (Kim et al., 2016).

NO dysregulation appears to be important in vascular tone regulation and PA remodeling in pulmonary arterial hypertension (PAH). Decreased NO availability is a common phenomenon in patients with PAH with impairment in the biosynthesis of NO (Klinger et al., 2013). In PAH, PA remodeling occurs along with changes in the molecular properties of smooth muscle cells (Schermuly et al., 2011). In cultured adult rat pulmonary arterial smooth muscle cells (PASMCs), downregulation of sGC and PKG expression occurred in parallel to phenotypic changes ranging from contractile to synthetic type, which might explain the PA remodeling in PAH (Lincoln et al., 2006). Interestingly, in a Sugen5416/hypoxia-induced PAH rat model, inhibition of NOS by NG-Nitro-L-arginine-methyl ester (L-NAME), revealed biphasic changes in NO availability, and decreased and increased in the early and late phase PAH, respectively. The latter change might counterbalance the excessive contractile tone of the chronic hypoxia-induced PAH arteries (Tanaka et al., 2017).

As a classical animal model of PAH, monocrotaline-injected rats (MCT-PAH) show typical medial thickening of PA with right ventricular hypertrophy. In PAH-MCT rats, a molecular biological investigation of PA tissue revealed increased expression of ROCK2 and decreased sGC (Lee et al., 2016). These changes may decrease the efficiency of the NO/cGMP pathway to enhance responsiveness to contractile agonists such as TXA_2_. Taken together with the vasoactive signals of TXA_2_ including eNOS activation in rat PA, we hypothesized that the putative downregulation of sGC in PAH-MCT might increase the responsiveness of PA to TXA_2_. However, the functional changes, including the sensitivity to the relevant pharmacological agents, were not rigorously investigated in the remodeled PA. In the present study, the concentration-dependent contractile responses of rat PA to U46619 were analyzed using a Mulvany-type isometric tension recording system. In addition, an immunoblot assay was performed to confirm and further elucidate the changes in the levels of TP receptor, eNOS, sGC, and ROCK in PA from PAH-MCT.

## Materials and Methods

### Animal Model

All experimental procedures were conducted with the approval of the Institutional Animal Care and Use Committee of Seoul National University (approval number: SNU-190408-3). Monocrotaline (Sigma, St. Louis, MO, United States) was dissolved in 2 mL of 1 M HCl and adjusted to pH 7.4 using 2 M NaOH solution. This aqueous solution was diluted to 17 mL with distilled water. Seven-week-old male Sprague-Dawley rats were randomly assigned and treated with a single intraperitoneal injection of monocrotaline (60 mg/kg) to induce the PAH-MCT model or an appropriate amount of saline. After 21 days, both the PAH-MCT rats and the age-matched control were sacrificed for further analysis, after measuring their body weight (b.w.).

### Arterial Tissue Preparation

Lung tissues were excised and stored in ice-cold normal Tyrode’s (NT) solution (140 mM of NaCl, 5.4 mM of KCl, 0.33 mM of NaH_2_PO_4_, 10 mM of HEPES, 10 mM of glucose, 1.8 mM of CaCl_2_, and 1 mM of MgCl_2_, pH 7.4 was adjusted with NaOH). The pulmonary artery bed was collected by removing the lung and bronchial tissues in NT solution. Excised pulmonary arteries were cleaned from perivascular adipose and other tissues under a microscope. Prepared arterial tissues were used for further analysis.

### Isometric Tension Recordings

Isometric tension was measured using a dual-wire multi-channel myograph system (620 M; DMT, Aarhus, Denmark). Excised arteries were cut into 2 mm arterial ring segments and mounted with 25 μm tungsten wire on an NT solution-filled organ chamber for tension recording. For stabilization, mounted arteries were rested in physiological salt solution (PSS; 118 mM of NaCl, 4 mM of KCl, 24 mM of NaHCO_3_, 1 mM of MgSO_4_, 0.44 mM of NaH_2_PO_4_, 5.6 mM of glucose, and 1.8 mM of CaCl_2_) for at least 15 min with a gas mixture (21% O_2_, 5% CO_2_, N_2_ balance) after a basal tone was applied. The whole experiment was maintained at 37°C. For the experiment, 80 mM of KCl-PSS was used to induce contraction (80K) for the evaluation of vessel integrity, and the dose-dependent response of U46619 was evaluated with the application of differential dosages from 1 to 200 nM.

### Histology

Pulmonary arterial ring segments were washed with phosphate-buffered saline (PBS) and fixed in 4% paraformaldehyde overnight. For histological analysis, paraffin-embedded tissues were cut and stained with hematoxylin and eosin (H&E) or Masson’s trichrome (MT). Digital images of the stained sections were obtained at 200 × magnification using the Aperio ImageScope 12.3 software.

### Western Blotting

Whole pulmonary arteries from the left lung were excised and homogenized with RIPA buffer (Millipore, United States) and protease/phosphatase inhibitor cocktail (Roche Diagnostics, Germany) for 1 h at 4°C. The samples were centrifuged at 13,000 ×*g* for 30 min at 4°C and the supernatant was collected. Protein concentration was determined using the Pierce^TM^ BCA Protein Assay Kit (Thermo Fisher Scientific, United States). The loading samples were prepared with Laemmli sample buffer, resolved by 8% SDS-PAGE, and transferred to polyvinylidene difluoride membranes in 25 mM of Tris, 192 mM of glycine, 0.01% SDS, and 20% methanol. Membranes were blocked in 1 × TBS containing 1% Tween-20 and 5% skim milk (blocking solution) for 1 h at room temperature with gentle rocking and incubated overnight at 4°C with relevant primary antibodies for detecting protein expression. Mouse anti-eNOS monoclonal antibody 1:1000 (Abcam, ab76198,United Kingdom), rabbit anti-sGC-α polyclonal antibody 1:1000 (Cayman, 160895, United States), rabbit anti-sGC-β1 polyclonal antibody 1:1000 (Cayman, 160897, United States), rabbit anti-PKG monoclonal antibody 1:1000 (Cell signaling, 3248, United States), rabbit anti-ROCK1 monoclonal antibody 1:1000 (Abcam, ab45171, United Kingdom), rabbit anti-ROCK2 polyclonal antibody 1:1000 (Abcam, ab71598, United Kingdom), rabbit anti-TXA_2_R polyclonal antibody 1:1000 (Abcam, ab233288, United Kingdom), and mouse anti-β-actin monoclonal antibody 1:10000 (Sigma, A1978, United States) were used. The signals were determined using ECL Plus Western blotting detection reagents (Amersham Biosciences, United Kingdom) and detected images were obtained by the Amersham^TM^ Imager 600 (Amersham Biosciences, United Kingdom). The intensity of each band was measured using the ImageJ analysis software.

### cGMP Concentration Measurement

cGMP concentration in the pulmonary arterial tissues was measured by a commercially available cGMP enzyme immunoassay (EIA) kit (Cayman). The excised pulmonary arteries were pulverized and homogenized with 5% trichloroacetic acid (TCA). The samples were centrifuged at 1,500 ×*g* for 30 min at 4°C and the supernatant was collected. The supernatants were analyzed according to the manufacturer’s instructions.

### Statistical Analysis

All data are expressed as mean ± SEM and the number of tested arteries is indicated as *n*. For all comparisons, arteries were obtained from at least three rats per protocol. Statistical differences between PAH-MCT and normal rats were analyzed using a two-sample T test. ANOVA was used to investigate more than two groups, and Tukey correction was performed for *post hoc* testing for significant differences. Two-way ANOVA was used to show the statistical differences in the concentration-dependent contractile response of U46619 between groups. Statistical differences were analyzed using SPSS Version 23 (IBM, SPSS, Chicago) and differences were considered when the *p*-value was less than 0.05.

## Results

### Increased Contractile Sensitivity to U46619 in the PA of PAH-MCT

**FIGURE 1:**
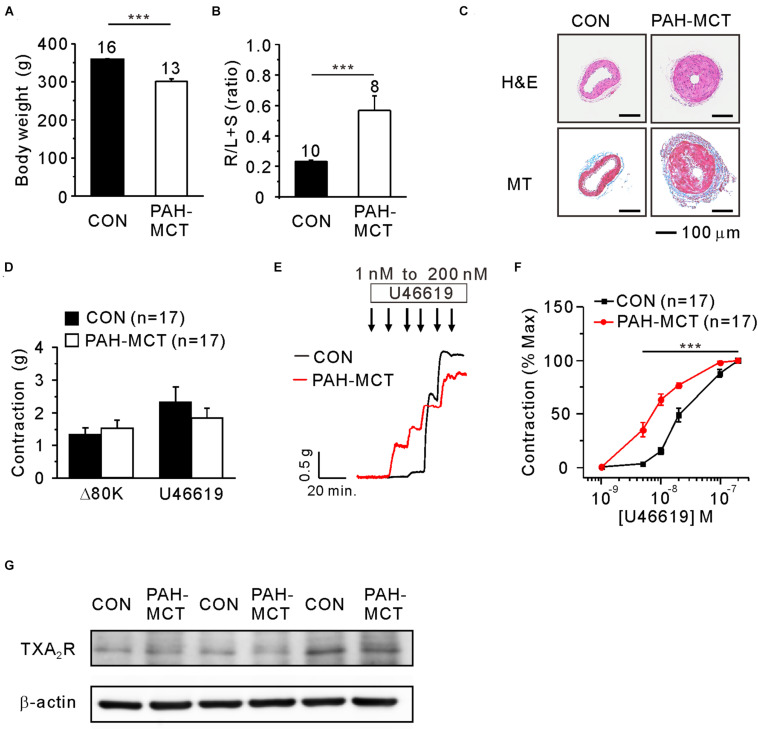
Increased contractile responsiveness of PA to TXA_2_ in PAH-MCT. Characteristics of PAH-MCT model with lower b.w. **(A)**, higher relative mass of the right ventricle (R/L + S mass ratio, **(B)**, and medial thickening of PA revealed by the H&E stained light microscopy [**(C)**, upper panel]. MT staining showed MCT-induced collagen deposition in PAH-MCT [**(C)**, lower panel, magnification of 200×. Scale bar: 100 μm]. Isometric tension recording of PA from CON and PAH-MCT rats. Contractile force of Δ80K and U46619 were represented with g **(D)**. The contractile responses to the stepwise increase in the concentration of U46619 **(E)**. The contractile responsiveness was analyzed with concentration-dependent contractile response curve [C-R curve, **(F)**]. The bar graphs represent the means ± SEMs. Statistical difference between groups are indicated as ***P < 0.001; bar graph with two sample *t*-test, and C-R curve with two-way ANOVA. **(G)** Representative three pairs of immunoblotting analyses comparing the expression of TP receptors in PAs between CON and PAH-MCT.

To characterize the PAH-MCT model, the b.w. changes, mass ratio of the right ventricle, and histological features of PA were analyzed. The b.w. on the 21st day after the MCT injection was lower in PAH-MCT than in CON (Figure 1A). The mass ratio of the right ventricles over the sum of the left ventricle and septum (RV/LV + S) was significantly higher in PAH-MCT than in CON (Figure 1B) (0.23 ± 0.006 vs. 0.57 ± 0.096; *p* = 0.0012). PA from PAH-MCT displayed a thicker medial layer than the PA from CON (Figure 1C, upper panel). MT staining revealed increased collagen deposition in the medial layer of PA from PAH-MCT (Figure 1C, lower panel).

In each vessel, the contractile response to 80 mM of KCl (80K-contraction) was initially measured. The PA of PAH-MCT and CON did not show a significant difference at 80K-contraction (Figure 1D). To analyze the sensitivity to U46619, the concentration-dependent increase in contractile tone was measured with the isometric tension recording system (Figure 1E). The maximum contraction induced by 200 nM of U46619 was higher than that induced by 80K-contraction in CON. The U46619-induced maximum contraction showed a tendency of smaller amplitude in PAH-MCT, and statistical significance was not found owing to the cases with sizable amplitude (Figure 1D). When normalized to the maximum U46619-contraction, the concentration-dependent contractile response curve (C-R curve) showed significantly higher sensitivity to U46619 in PAH-MCT than in CON. Notably, the contractile response at relatively low concentrations (5–20 nM) was more significant in PAH-MCT than in CON (Figure 1F). Despite the higher sensitivity to U46619 in PAH-MCT, the expression of TXA_2_ receptor (TP) was not different between CON and PAH-MCT (Figure 1G).

### Decreased Expression of NO/cGMP-Regulating Proteins and Increased Expression of ROCK2 in the PA of PAH-MCT

**FIGURE 2:**
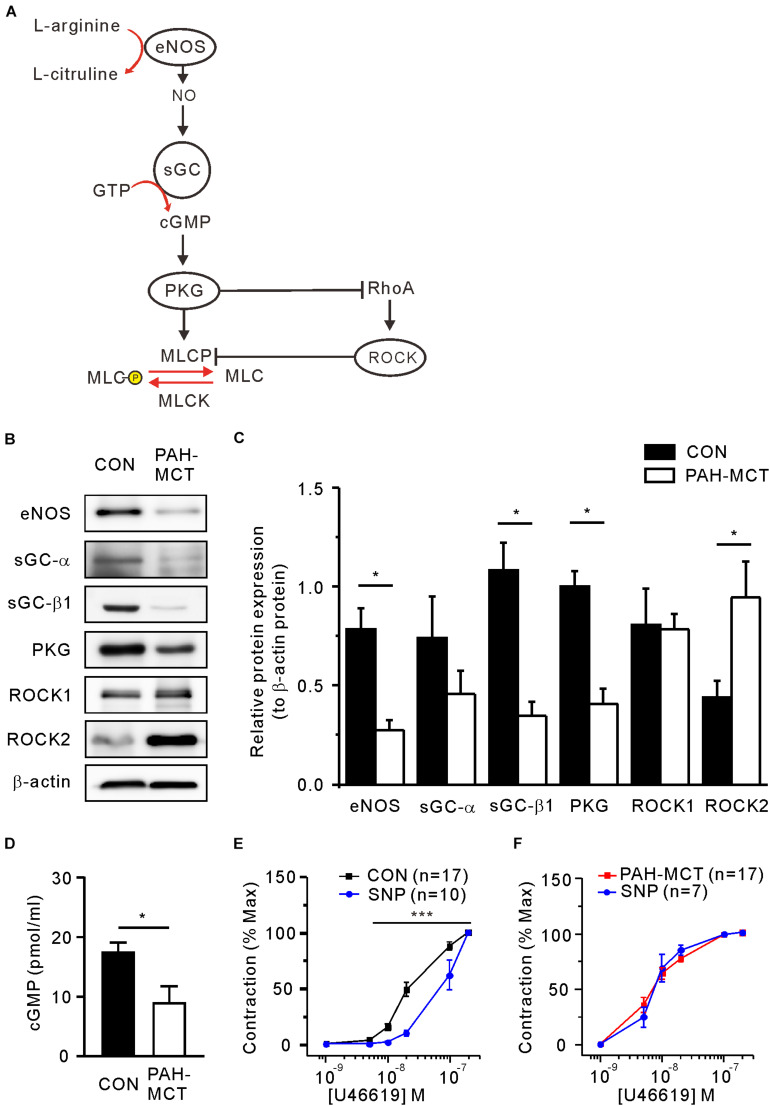
Altered expression of NO/cGMP-regulating enzymes and ROCK in PAH-MCT. **(A)** Schematic drawing of NO/cGMP pathway molecules involved in the regulation of vascular smooth muscle contractility. **(B)** Representative western blot images of NO-sGC-cGMP pathway molecules with β-actin as a standard control. **(C)** Results of band intensity relative to β -actin (*n* = 4, respectively). **(D)** cGMP levels of PA tissues measured with EIA (*n* = 4). *P < 0.05 vs. PAH-MCT; Mann-Whitney *U* test. **(E,F)** Effect of SNP (30 μM on the C-R curves of CON **(E)** and PAH-MCT **(F)**. ***P < 0.001; two-way ANOVA.

The sGC and PKG are known to mediate the relaxing signal from eNOS and NO to downstream proteins such as myosin light chain phosphatase (MLCP) and ROCK (Figure 2A). The immunoblot study showed decreased eNOS expression in PA of PAH-MCT (Figure 2B). sGC is a heterodimer composed of one alpha (sGC-α) and one heme-binding beta subunit (sGC-β). Immunoblot analysis of sGC showed a tendency of lower expression in PAH-MCT whereas statistical significance was found in the level of sGC-β1 (Figures 2B,C). The expression of PKG was also lower in PAH-MCT than in CON (*p* = 0.030). In contrast, the expression of ROCK2 was increased in PAH-MCT (*p* = 0.043), while the level of ROCK1 was not changed (Figure 2C). cGMP level in the PA tissues was lower in PAH-MCT than in CON (Figure 2D).

To investigate the contribution of relaxing signals distal to NO in the U46619-induced contraction, we analyzed the C-R curve after pretreatment with the NO donor sodium nitroprusside (SNP, 30 μM, 10 min). In the CON PA, the C-R curve was shifted to the right by SNP, implying lowered sensitivity to U46619 (Figure 2E). In contrast, the pretreatment with SNP did not affect the C-R curve in PAH-MCT (Figure 2F).

### Pharmacological Regulation of eNOS, sGC, and ROCK in PAH-MCT and CON

**FIGURE 3:**
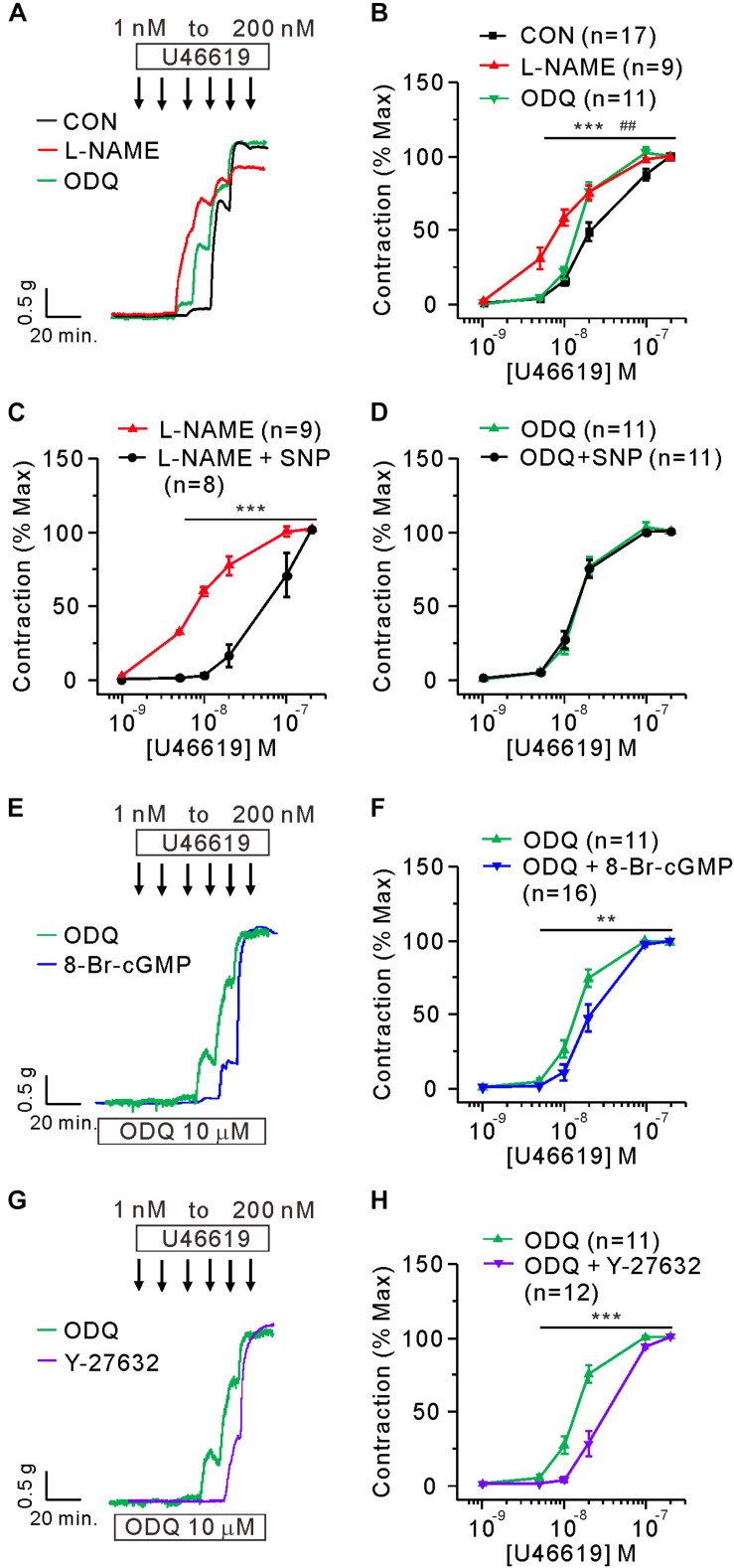
Mimicking the functional changes in PAH-MCT by the inhibitors of eNOS and sGC in the PA of CON. **(A,B)** Effect of preincubation with L-NAME (100 μM) and ODQ (10 μM) on the C-R curves of CON. ***P < 0.001 L-NAME vs CON and ##*P* < 0.01 ODQ vs CON; two-way ANOVA. **(C,D)** Effect of SNP (30 μM on the sensitized C-R curves by L-NAME **(C)** or by ODQ **(D)**. **(E,F)** Effect of 8-Br-cGMP (30 μM) on the sensitized contraction and the C-R curve shifted by ODQ. **(G,H)** Effect of Y-27632 (10 μM) on the sensitized contraction and the C-R curve shifted by ODQ. Statistical difference between groups are indicated with **P < 0.01 or ***P < 0.001; two-way ANOVA.

To mimic the downregulation of eNOS and sGC proteins in the PAH-MCT, the PA of CON was pretreated with NOS inhibitor (L-NAME, 100 μM) or sGC inhibitor (ODQ, 10 μM). Both pharmacological inhibitors increased the sensitivity of PA to U46619 in CON, and the effect of L-NAME was relatively more prominent (Figures 3A,B). In the presence of L-NAME, the exogenous NO donor (SNP, 30 μM) shifted the C-R curve to the right, while SNP had no effect on the C-R curve in the presence of ODQ (Figures 3C,D). We compensated for sGC inhibition by using 8-bromoguanosine 3′, 5′-cyclic monophosphate (8-Br-cGMP), a membrane permeable analog of cGMP. The additional pretreatment with 5 μM of 8-Br-cGMP shifted the C-R curve to the right in the presence of ODQ (Figures 3E,F). Since the inhibition of ROCK2 was a plausible activity of PKG in the PA myocytes, we evaluated the effect of the ROCK inhibitor Y-27632. The additional pretreatment with 10 μM of Y-27632 also shifted the C-R curve to the right in the presence of ODQ (Figures 3G,H).

**FIGURE 4:**
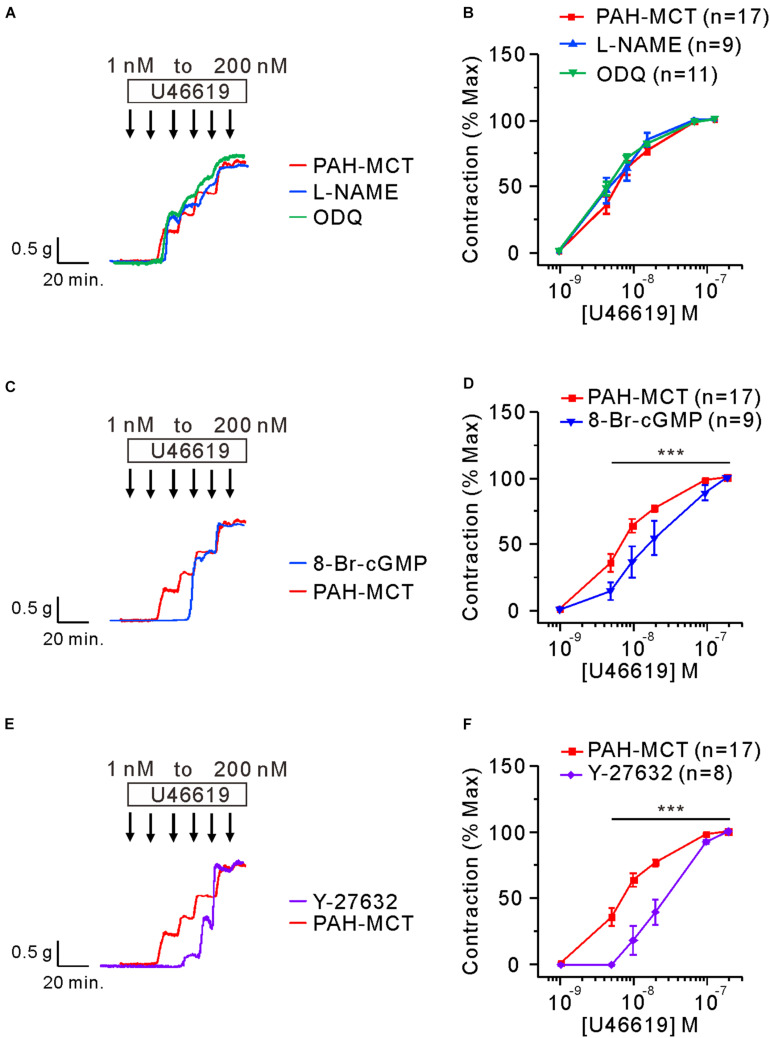
Effects of the inhibitors of eNOS, sGC, and ROCK on the C-R curves of U46619 in the PA of PAH-MCT. **(A,B)** No significant effect of pretreatment with L-NAME (100 μM) and ODQ (10 μM) on the C-R curves of PAH-MCT. **(C,D)** Rightward shift of the C-R curve by 8-Br-cGMP (30 μM). **(E,F)** Rightward shift of the C-R curve by Y-27632 (10 μM). Statistical difference between groups are indicated with ***P < 0.001; two-way ANOVA.

Next, the effects of the above pharmacological agents were tested in the PA of PAH-MCT. In contrast to the PA of CON, neither the inhibition of eNOS (L-NAME) nor sGC (ODQ) had an effect on the C-R curve of U46619 (Figures 4A,B). Despite the insignificant effect of L-NAME and ODQ, pretreatment with 8-Br-cGMP shifted the C-R curve to the right in the PA of PAH-MCT (Figures 4C,D). In addition, pretreatment with Y-27632 shifted the C-R curve to the right (Figures 4E,F).

**FIGURE 5:**
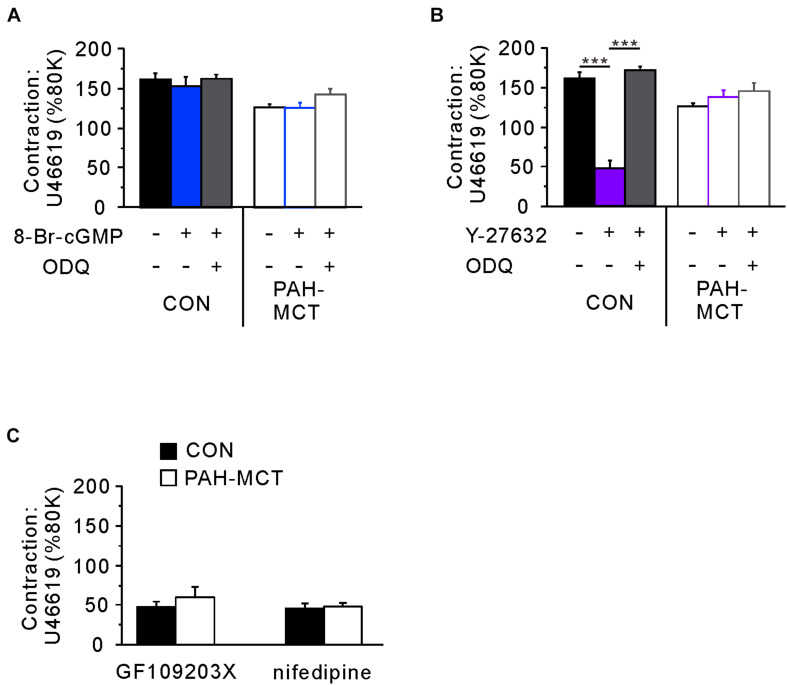
Comparative analyzes of the signaling pathways determining the maximum tone of PA induced by 200 nM of U46619. **(A)** The pretreatment with 8-Br-cGMP alone or with ODQ did not affect the maximum contraction normalized to the 80K-contraction in each PA from CON and PAH-MCT. **(B)** Different effects of the pretreatment with Y27632 on the maximum tone of CON and PAH-MCT. Note that the inhibition by Y27632 was prevented by the co-treatment with ODQ in CON while not altered in PAH-MCT. **(C)** Effects of GF109203X (10 μM) and nifedipine (1 μM) on the maximum contraction induced by 200 nM of U46619. The bars represent the means ± SEMs. Statistical difference between groups are indicated as ***P < 0.001. Comparison of the effects of 8-Br-cGMP and Y27632.

Since the C-R curves shown above are normalized values (% contraction induced by 200 nM of U46619), the pretreatment with vasodilators such as 8-Br-cGMP and Y-27632 affected the maximum tone in the tested vessels. Since the initial 80K contraction was measured in the absence of the pharmacological agents, we compared the normalized maximum contraction (% of 80K contraction) in each tested condition (Figure 5). To investigate this, a single dose of U46619 (200 nM) was applied in the absence or presence of 8-Br-cGMP and ODQ. Pretreatment with 8-Br-cGMP did not affect the maximum contraction induced by U46619. Furthermore, the combined treatment with ODQ (8-Br-cGMP + ODQ) did not have a significant effect on maximum contraction (Figure 5A).

In contrast to the effect of 8-Br-cGMP, the direct inhibition of ROCK (Y-27632) largely suppressed the maximum contraction by U46619. However, when pretreated with both ODQ and Y-27632, the maximum contraction was restored to the control level in the PA from CON (Figure 5B, left panel). Interestingly, in the PA from PAH-MCT, neither pretreatment with Y-27632 alone nor the co-treatment with ODQ affected the maximum contraction by U46619 (Figure 5B, right panel).

The resistance to Y-27632 in the maximum contraction of PAH-MCT might be due to a hidden compensatory upregulation of contractile signaling pathways other than ROCK, such as voltage-operated L-type Ca^2+^ channels (VOCC_L_) or protein kinase C (PKC). To test this possibility, we examined the effect of the VOCC_L_ inhibitor (nifedipine, 1 μM) and an inhibitor of PKC (GF109203X, 10 μM). Both inhibitors decreased the maximum contraction by around 70% in PAH-MCT as well as in CON (Figure 5C).

## Discussion

**FIGURE 6:**
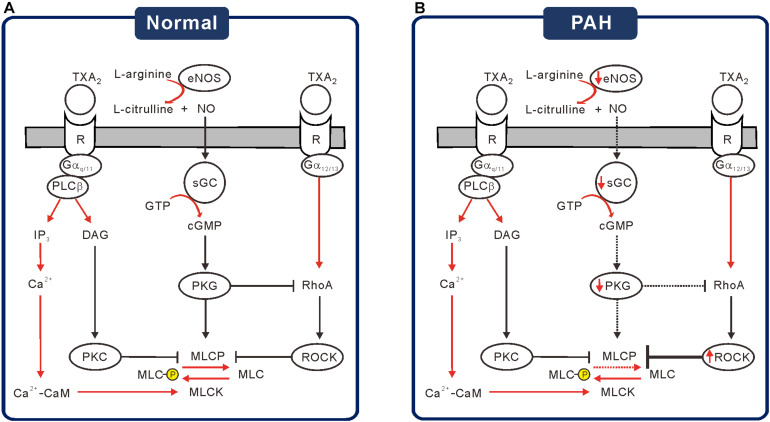
Schematic diagrams of the altered signaling pathways suggested for the increased TXA_2_ sensitivity in PAH-MCT. **(A)** Under the basal or stimulated signaling through NO-sGC-cGMP-PKG cascade, PKG activates MLCP but inhibits ROCK to prevent excessive contraction by TXA_2_. **(B)** Under the pathological condition of PAH, decreased signaling via NO-sGC-cGMP-PKG would increase the MLC phosphorylation, resulting in increased responsiveness to TXA_2_.

The present study showed that the PA segments from rat with PAH-MCT are contracted by nanomolar ranges of the TXA_2_ analog, which could be accounted for by the downregulation of NO-related signaling enzymes (eNOS, sGC, and PKG) and by the upregulation of ROCK2 (Figure 6). Although the membrane localization of TP receptors was not analyzed, the immunoblot analysis did not show changes in TP receptor expression (Figure 1G). Consistent with the schematic summary of the changes in PAH-MCT (Figure 6), the pharmacological inhibitors of eNOS and sGC shifted the C-R curves of CON PA in the rightward direction, but not in the PA from PAH-MCT (Figures 3, 4). In PAH-MCT, the inhibition of ROCK effectively restored the C-R curve similar to that in CON (Figure 4F). Although the PKG level in PA was downregulated in PAH-MCT, the application of the PKG activator 8-Br-cGMP reversed the increased responsiveness to U46619 in PAH-MCT (Figure 4D). Taken together with the results of the immunoblotting assay, the analysis of the C-R curves appears to be an appropriate approach to reveal the functional implication of the changes in the intracellular signals in PASMCs.

TP receptor signaling in the arterial contraction is mediated through Gα_12/13_ to RhoA/ROCK or through Gα_q/11_ to PLCβand InsP_3_/diacylglycerol (DAG)/PKC signaling pathways. It is generally known that activated ROCK induces MLC2 phosphorylation indirectly, which is mediated by the inhibitory phosphorylation of Thr residues (T696 and T853) of MYPT that also prevent the stimulatory phosphorylation of neighboring Ser residues (S695 and S852) (Butler et al., 2013). MYPT1 is a member of the myosin phosphatase targeting protein (MYPT) family consisting of five genes, MYPT1, MYPT2, MBS85, MYPT3, and TIMAP, which function as targeting and regulatory subunits to confer substrate specificity and subcellular localization on the catalytic subunit of type 1δ protein serine/threonine phosphatase (PP1cδ) (Grassie et al., 2011). Taken together, we propose that the upregulation of ROCK along with downregulated sGC/PKG in PAH-MCT are responsible for the sensitized contractile response to TXA_2_.

It was notable that the maximum contraction induced by 200 nM of U46619 was not affected by SNP or 8-Br-cGMP. In contrast, the inhibition of VOCC_L_ and PKC significantly reduced the maximum contraction (Figures 5A,C). These results suggested that NO/cGMP-dependent signals have a modulatory role in MLC phosphorylation rather than a direct inhibition of Ca^2+^ signals and contractile mechanisms elicited by TP stimulation. The inhibition of ROCK showed differential effects on maximum contraction; Y-27632 significantly suppressed the maximum contraction in CON, but not in PAH-MCT. Interestingly, even in the PA of CON, Y-27632 did not decrease the maximum contraction in the presence of ODQ (Figure 5B). The results suggested a dominant relaxing signal from cGMP-PKG rather than a contractile signal via ROCK that converges on MYPT.

The phosphorylation states of Ser695/Ser852 and Thr696/Thr853 have mutually antagonistic effects on MYPT activity (Qiao et al., 2014). PKG phosphorylates and activates the Ser residues and induces translocation to MLC2, the dephosphorylation of which promotes vasorelaxation. In contrast, ROCK and other pro-contractile signals increase the phosphorylation of Thr residues in MYPT which induces membrane translocation and lowers the chances of interaction with MLC2 (Butler et al., 2013). In this scheme, the deficiency in cGMP-PKG signaling (e.g., ODQ treatment) would not allow for the control of MLC2 by MYPT, and the inhibitory effect of Y-27632 by preventing the interaction between MYPT and MLC2 could not be demonstrated. Since the downregulated eNOS/sGC/PKG in the PA of PAH-MCT would be equivalent to pharmacological inhibition, the effect of Y-27632 was not observed, especially in the conditions of maximum contraction.

Taken together, our present results could be interpreted based on the schematic model of the antagonistic influence on the MLCP activity between the sGC/cGMP/PKG and RhoA/ROCK pathways (Figure 6). The downregulation of sGC and PKG possibly minimized the role of MLCP, which is also the target of ROCK. Therefore, the pharmacological inhibition of ROCK might not affect the maximum level of contraction, while still shifting the C-R curve. Since the relative contribution from the other contractile signaling pathways was not altered in the PA from PAH-MCT (Figure 5C), we cautiously emphasized the critical counteractive regulatory effects of the eNOS-sGC-PKG and RhoA-ROCK pathways on MLCP in PA contraction by TP receptor stimulation. The altered expression of the above signaling molecules in the PASMCs would result in the dysregulation of vasodilation activity (Figure 6B).

The immunoblot assay revealed a significant decrease of eNOS expression in the PA of PAH-MCT. Downregulation of eNOS vascular endothelium is one of the major pathophysiological findings in cardiovascular diseases (Bauersachs et al., 1998; Chou et al., 1998; Kobayashi et al., 1999; Li et al., 2002; Lee et al., 2016). It is generally known that endothelial eNOS is a major source of NO which diffuses to neighboring tissues to activate signaling cascades, including sGC. In rat PA, we have previously reported that the medial layer, most likely the smooth muscle cells also express eNOS. Although the level of eNOS expression in the PA medial layer is minute compared to the endothelium, TP stimulation could induce the muscular eNOS phosphorylation to partly contribute to the relaxing signals intrinsic to the myocytes (Kim et al., 2016). However, in the present study, the C-R curves were obtained in PA with an intact endothelium since the process of endothelium removal by mechanical abrasion generally induces unstable responses during U46619-induced contraction experiments. Considering the relatively minute expression of eNOS in the medial layer of PA, the decreased eNOS expression in the PAH-MCT would mostly reflect the downregulation in the endothelium (Figure 2).

Along with eNOS, the downregulation of the sGC subunit, especially sGC-β1, was a prominent and consistent finding in PAH-MCT. The critical role of sGC in the regulation of vascular contractility could be demonstrated by the effect of ODQ. In this respect, the abolished influence of ODQ treatment on the C-R curve of PA from PAH-MCT (Figure 4B) suggested that the downregulation of sGC was functionally crucial and could be underestimated when simply interpreted from the decreased expression level (Figure 2C). The downregulation of sGC and the decreased cGMP (Figure 2D) observed in the present study could be consistent with the rationale for the clinical application of PDE5 inhibitors for the treatment of PAH. In addition, a direct activator of sGC, riociguat, has been recently introduced for clinical studies (Montani et al., 2014). The concomitant partial decrease in PKG expression should be considered to interpret the functional changes under pathological conditions. Decreased PKG expression has also been reported in primary aortic vascular smooth muscle cells treated with inflammatory cytokines (Browner et al., 2004; Choi et al., 2018).

ROCK2 expression was increased in PA from PAH-MCT and the C-R curve was restored with ROCK inhibition. The pathophysiological role of ROCK-dependent signaling has also been found in systemic arteries. In the mesenteric artery of spontaneous hypertensive rats, H_2_O_2_ induces c-Src-dependent TXA_2_ release and provokes vascular contractile responses through multiple signaling pathways, including ROCK (García-Redondo et al., 2015). Consistent with the pathophysiological implication of the TXA_2_ pathway, both genetic and pharmacological suppression of TXA_2_/TP signaling confers microvascular protection against oxidative injury in mesenteric arteries (Chiang, 2019).

In addition to the changes in PA contractility, increased synthesis of TXA_2_ and local availability might also underlie the higher tension under pathological conditions. Experimentally, it was reported that a direct injection of U46619 induced a severe prolonged increase in systolic blood pressure, and death of the tested mice, which was not observed in the TP knockout mice (Sparks et al., 2013). Clinically, there is evidence that increased TXA_2_ synthesis possibly contributes to increased vascular resistance in children with pulmonary hypertension and pregnancy-induced hypertension (Fitzgerald et al., 1990; Adatia et al., 1993). PAH patients showed higher TXA_2_ synthesis with decreased prostacyclin (PGI_2_) (Christman et al., 1992; Liel et al., 1993; Kazimierczyk and Kamiński, 2018) and platelet aggregation (Nakonechnicov et al., 1996; Varol et al., 2011), and increased thrombosis is one of the criteria for PAH (Farber and Loscalzo, 2004). Despite the repeatedly observed pathophysiological role of TXA_2_ in PAH, the pharmacological antagonist of the TP receptor has not been clinically investigated yet. Instead, the administration of PGI_2_ analog is one of the options for PAH treatment (Montani et al., 2014).

## Conclusion

During the pathological condition of PAH, the combined increase in TXA_2_ availability and contractile sensitivity to TXA_2_ may worsen the burden of pulmonary circulation. Along with the functional changes, PA wall thickening dramatically increased peripheral resistance and right ventricular afterload in PAH. The markedly lowered threshold concentration of U46619 suggested vulnerability to collapse or uneven regional perfusion in the PAH lung. Since the C-R curves and their changes by pharmacological agents are consistent with the molecular biological findings than the simple comparison of maximum contraction levels, our present study emphasizes the implication of physiological parameters obtained from myography studies.

## Data Availability Statement

The original contributions presented in the study are included in the article/supplementary material, further inquiries can be directed to the corresponding author/s.

## Ethics Statement

The animal study was reviewed and approved by the Institutional Animal Care and Use Committee (IACUC) of Seoul National University.

## Author Contributions

SC and SK: conception and design of study. SC, HN, and RV: conducting experiments, acquisition of data, analysis, and interpretation of data. SC, HY, and HK: drafting the manuscript. SK: revising the manuscript critically for important intellectual content. All authors contributed to the article and approved the submitted version.

## Conflict of Interest

The authors declare that the research was conducted in the absence of any commercial or financial relationships that could be construed as a potential conflict of interest.
